# Managements for perioperative anxiety in patients with gastrointestinal cancers

**DOI:** 10.3389/fpsyt.2024.1391403

**Published:** 2024-10-21

**Authors:** Ying Li, Juan Du, Li Du, Shan Li, Jianping Zhang

**Affiliations:** ^1^ Department of Anesthesia Surgery, Taizhou Central Hospital (Taizhou University Hospital), Taizhou, China; ^2^ Health Examination and Oncology Screening Center, Chongqing University Cancer Hospital, Chongqing, China; ^3^ Educational Administration Department, Chongqing University Cancer Hospital, Chongqing, China; ^4^ Department of Anesthesia Surgery, Mianyang Central Hospital, Mianyang, China; ^5^ Department of General Surgery, The Affiliated Hospital of Jiujiang University, Jiujiang, China

**Keywords:** gastrointestinal cancers, perioperative anxiety, nursing intervention, psychological disorder, management

## Abstract

Gastrointestinal (GI) cancers are the most common malignancies, while surgical intervention remains the sole therapeutic approach offering the possibility of a definite cure for cancer. Perioperative anxiety negatively impacts the recovery of GI cancers. Recently, mounting studies have demonstrated that proper nursing interventions may alleviative perioperative anxious illnesses in patients with GI cancers. We conducted a first comprehensive review to summarize all the current evidence on this topic. After a systematically search in the six common databases, eighteen relevant studies were included for further analysis. The present review highlighted that there is a high prevalence of perioperative anxiety in patients with GI cancers (e.g., colorectal cancer, gastric/stomach cancer, hepatocellular carcinoma, gallbladder cancer, and esophageal cancer), while specific nursing interventions are the reliable methods to reduce postoperative anxiety. These nursing strategies include, but are not limited to, therapeutic listening intervention, implementing perioperative music, predictive nursing, progressive relaxation exercises, psychological interventions in the nursing care, comprehensive nursing, continuous nursing care, video-based nursing education, multidisciplinary cooperative continuous care, accelerated rehabilitation nursing, TCM nursing, evidence-based early warning nursing, target nursing care, and high-quality nursing. Since several limitations existed in the eligible studies as well as in this review, a well-designed multicenter RCT with large sample size is still warranted for the confirmation of nursing intervention for managing perioperative anxiety in patients with GI cancers. Also, future studies should focus on the long-term effects of relevant interventions, specific patient populations, multidisciplinary approaches, technological innovations, and educational programs.

## Introduction

Gastrointestinal (GI) cancers are the most common malignancies, accounting for over one-quarter (26%) of all cancers worldwide. In addition, they are responsible for approximately one-third (35%) of all cancer-related mortality (1). Currently, a growing elderly population is expected to result in further increases in incidence of GI cancers in the coming decades (2). It is predicted that the global number of new cases of GI cancers may increase by 58% (7.5 million) in 2040. The types of GI cancers include the malignancy of colorectum (approximately 1.8 million new cases in 2018), stomach (1 million cases), livers (840, 000 cases), esophagus (570, 000 cases), pancreas (460, 000 cases), gallbladder, biliary tract, small intestine, and anus (1). The common risk factors for GI cancers include alcohol consumption, tobacco smoking, chronic infection, diet, and obesity (3). Despite the facts that systemic treatments of GI malignancies have improved significantly in the last few decades, surgical intervention remains the sole therapeutic approach offering the possibility of a definite cure for cancer (4). Aside from the decision to undergo surgery and the option of surgical techniques, perioperative treatments (i.e., neoadjuvant chemotherapy, radiotherapy, and preparing measures for surgery) are also crucial to achieve the desired outcomes (5).

In the recent years, to ensure satisfying postoperative outcomes for patients with GI cancers, it needs to be tailored to the features of both the patient and the operation (6). Perioperative anxiety is a significant concern for patients with GI cancers. As these patients face the daunting prospect of surgery and its associated uncertainties, managing anxiety becomes crucial not only for their psychological well-being but also for the success of the surgical procedure and subsequent recovery. It is common for patients to experience anxiety and fear when being admitted to the hospital for surgery. As a result, surgical procedures and hospitalization can pose a threat to patients due to the situation for physical changes and psychological reactions (7). What’s more, patients scheduled for oncologic surgery face an even more challenging situation due to malignancy is a cause of clinically significant suffering (8). It is well known that perioperative anxiety negatively impacts recovery for patients, which may result in harmful effects after surgery, such as increased use of anesthetics, heightened pain during and after operations, and prolonged hospitalization (9). Therefore, the ability to manage anxiety symptoms and negative emotional reactions is crucial for the recovery and the quality of life of cancer patients. People with cancer may benefit from psychotherapeutic interventions in terms of reducing emotional distress (10). Thus, more attention should be paid to the perioperative period of patient’s psychosocial diseases, such as anxiety. Perioperative anxiety may be multidimensional, including physiological impacts (i.e., a series of stress reactions), psychological disorders, social influences (i.e., family support, economic pressures, and work impact). In terms of psychological dimensions, several psychopathological characteristics often related to alexithymia or state-trait anxiety with different neurobiological underpinnings (11, 12). Based on these facts, perioperative anxiety is a complex problem that requires comprehensive interventions from multiple dimensions to help patients better cope with the stress of surgery and promote postoperative recovery.

Managements of perioperative anxiety in patients with GI cancers involve a variety of approaches, including psychological interventions (i.e., cognitive-behavioral therapy, supportive psychotherapy, and nursing interventions), pharmacological interventions (i.e., anxiolytics, beta-blockers), preoperative education, and relaxation techniques. Mounting studies have demonstrated that proper nursing can provide effective perioperative interventions to help cancer patients deal with anxiety (13). Nevertheless, during clinical practice, nurses commonly focus on physical health and underemphasize psychological issues. Besides, there is a lack of systematicity and standard methods in nursing intervention related to patient’s anxious illnesses. In the recent years, various nursing interventions have emerged gradually to alleviative perioperative anxiety. At present, however, different studies use a variety of assessment tools for measuring anxiety and different interventions for managing perioperative anxiety in GI cancers, making it difficult to compare results and establish clear guidelines. Therefore, we performed a comprehensive review to collect the published data on this topic, which might be useful in guiding clinical therapeutic decisions. For the first time, we conducted a comprehensive review to summarize all the current evidence on the topic of the different interventions on perioperative anxiety in patients with GI cancers, which might help guide clinically applicable specifications for these sufferers.

## Methods

In order to detect the eligible studies related to object of this review, six electronic databases (i.e., MEDLINE [PubMed], web of science, Cochrane Library, Google Scholar, EMBASE, and PsychINFO) were consulted. The search was systemically retrieved up to September 1, 2023. Search strategies applied to screen the qualified publications in the MEDLINE database were: ((((((((((((((((((((((((“Gastrointestinal Neoplasms”[Mesh]) OR (Gastrointestinal Neoplasm)) OR (Neoplasm, Gastrointestinal)) OR (Neoplasms, Gastrointestinal)) OR (Cancer of Gastrointestinal Tract)) OR (Gastrointestinal Tract Cancer)) OR (Gastrointestinal Tract Cancers)) OR (Cancer of the Gastrointestinal Tract)) OR (Gastrointestinal Cancer)) OR (Cancer, Gastrointestinal)) OR (Cancers, Gastrointestinal)) OR (Gastrointestinal Cancers)) OR (gastric cancer)) OR (rectal cancer)) OR (colon cancer)) OR (colorectal cancer)) OR (Esophageal cancer)) OR (liver cancer)) OR (Pancreatic cancer)) OR (Gallbladder cancer)) OR (biliary tract cancer)) OR (small intestine cancer)) OR (anal cancer)) AND (((((((((“Anxiety”[Mesh]) OR (Angst)) OR (Social Anxiety)) OR (Anxieties, Social)) OR (Anxiety, Social)) OR (Social Anxieties)) OR (Hypervigilance)) OR (Nervousness)) OR (Anxiousness))) AND (((perioperative) OR (postoperative)) OR (preoperative)). Besides, we also manually inspected the reference list of the related articles to identify more eligible studies. The following types of publications were excluded, including studies with duplicate data, review, letters, comments, meeting abstracts, case reports, and experimental experiments.

Two authors independently conducted the process of searching. The criteria for study inclusion were set to include any study designs related to the research topic, English language studies without publication date restrictions, and the availability and accessibility of the relevant studies. If ambiguities arising, it could be resolved by a third author or the corresponding author. In order to extract the key data, a standardized table of data collection was used. The following information were extracted, including the first author’s name, study area, year of publication, study design, population size of the participants, assessment for anxiety, specific nursing methods and protocols, and the main findings of the included study.

### Literature search and eligible study characteristic

**Figure 1:**
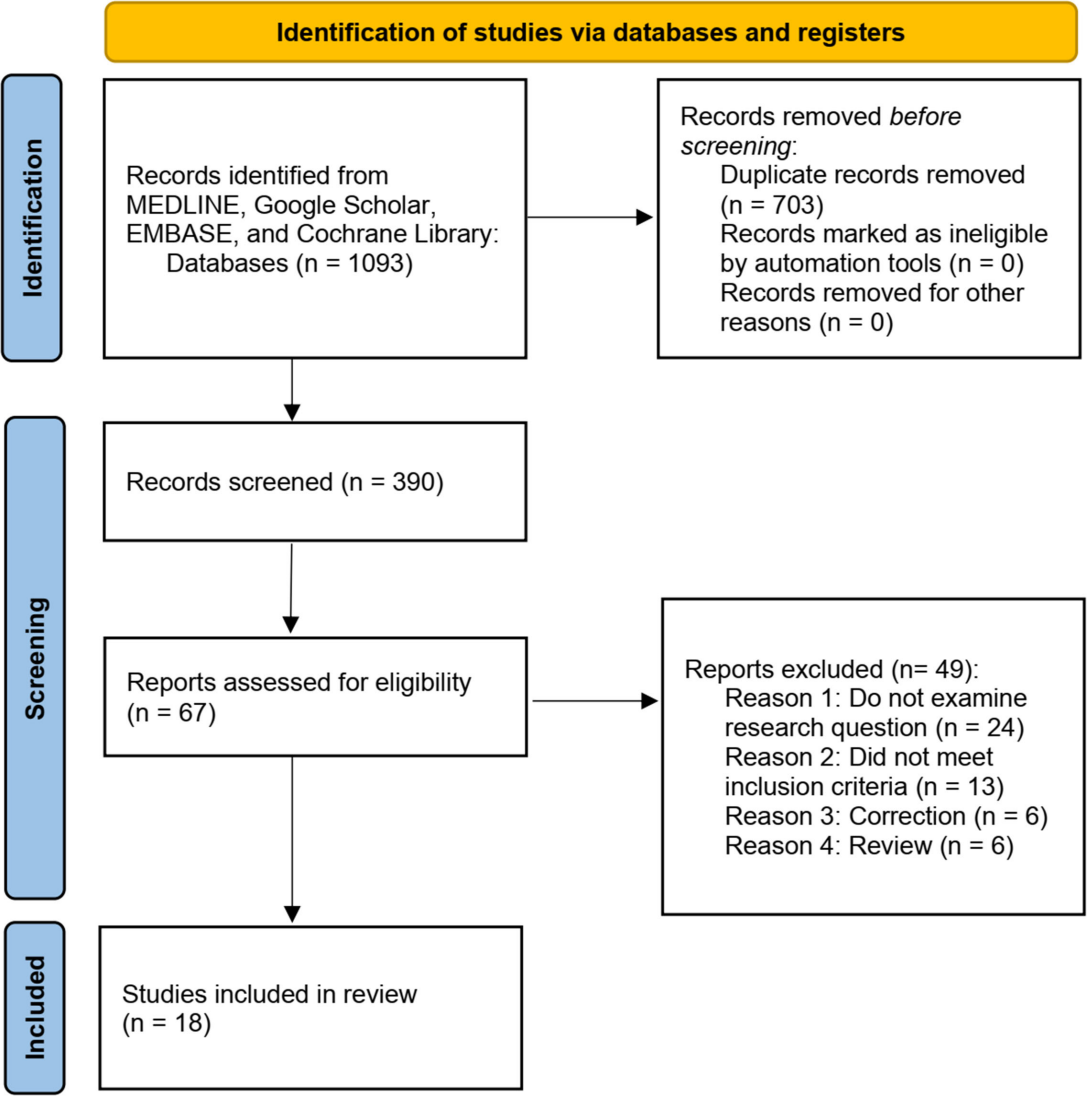
The PRISMA flow diagram.

As shown in **Figure 1**, the PRISMA flow diagram was used to identify relevant studies on the issue of nursing intervention and perioperative anxiety disorder in patients with GI cancers. The initial database search yielded 1093 records, of which 432 from MEDLINE, 272 from Google Scholar, 221 from Embase, and 168 from the Cochrane Library. After title and abstract screening, 684 duplicates were removed. Through the full-text screening of 87 studies, 69 studies were further eliminated. Based on the predefined inclusion criteria, eighteen relevant studies (14–31) were considered to be eligible. Those studies were published from 2021 to 2023. The geographical location of the studies included Brazil, Netherlands, China, and Turkey. The sample size of these studies ranged from 50 to 155. The study design included randomized controlled trial, cohort, case-control, prospective study, and retrospective study. Based on an exhaust search, the GI cancer types included colorectal cancer (9 studies), gastric cancer (5 studies), liver cancer (2 studies), gallbladder cancer (1 study), and esophageal cancer (1 study). The assessments for anxiety included State-Trait Anxiety Inventory (TAI), Hospital Anxiety and Depression Scale (HADS), Self-rating anxiety scale (SAS), and Hamilton anxiety scale (HAMA). The nursing interventional methods included therapeutic listening intervention, measurements that ascertaining and meeting personalized psychosocial needs, implementing perioperative music, predictive nursing, progressive relaxation exercises. **Tables 1**–**3** listed the characteristics of the 18 included studies. The main findings of the eighteen eligible studies were summarized and discussed in the following sections.

**Table 1:** Characteristics of the included studies on colorectal cancer.

Study/Reference	Study design	Sample size	Cancer type	Assessment for anxiety	Nursing interventional methods and protocols	Main findings
(14) Brazil	RCT	50 patients	Colorectal cancer	STAI	Therapeutic listening intervention. Protocol: In the preoperative period, patients had half-hour to talk to the investigators about their experience with hospitalization for cancer treatment.	The therapeutic listening intervention did not cause differences on the state anxiety and surgical fears compared to the control group.
(15) China	Longitudinal study	67 patients	Colorectal cancer	HADS	Ascertaining and meeting personalized psychosocial needs. Protocol: NA.	Nearly 50.0% of participants were identified to have anxiety disorder before the surgery, while the number decreased to 37.3% in postoperative period. The different was significant (P<0.05). Age and educational background were the significant variables that influenced anxiety before and after surgery.
(17) China	Cohort	130 patients	Rectal cancer	SAS	Predictive nursing. Protocol: Preoperatively, the patients and their families were educated about rectal cancer; evaluating the patients’ psychological states and eliminating their anxiety; multimedia mode was applied to explain stoma care, diet indexes, bathing mode, and exercise mode.	The SAS scores were significantly lower in the predictive nursing group than the control group (P < 0.05).
(27) Turkey	RCT	63 patients	Colorectal cancer	STAI	Progressive relaxation exercises. Protocol: The progressive relaxation exercises were applied in patients for 15min preoperatively and on postoperative days 1, 2, and 3 after breathing exercise training.	As compared to the routine care, progressive relaxation exercises could significantly reduce postoperative pain, the rate of using opioid analgesic, and anxiety levels on postoperative day 0 (all P<0.05).
(21) China	Cohort	120 patients	Rectal cancer	SAS and HAMA	Psychological interventions in the nursing care. Protocol: 1. Preoperative psychological care; 2. Provided regular cleaning stoma; 3. Guided the patients could manage their stomas independently; 4. Diet and nutrition education; 5. Individualized psychological intervention; 6. Home care was taught before the discharge.	The SAS and HAMA scores in the study group were significantly lower than the control group (all P < 0.05). Psychological interventions in the nursing care could alleviate the patients’ bad moods, improve the quality of life, elevate the sleep durations.
(22) China	Cohort	110 patients	Colon cancer	HAMA	Comprehensive nursing. Protocol: 1. Preoperative nursing: understood the patient’s physical state, medical and surgical history, systematic health education and psychological counseling; 2. Intraoperative nursing: cooperated with physicians in performing surgery-related nursing during the surgery; 3. Postoperative nursing: set comfortable position after surgery, appropriate exercise, and rigorous care.	The HAMA scores in the study group were significantly lower than those in the control group (P<0.05). Comprehensive nursing could significantly reduce the postoperative complication rate (9% vs 25%), increase the gastrointestinal quality of life index (GIQLI) score, and improve the prognosis.
(23) China	RCT	60 patients	Colorectal cancer	SAS	Comprehensive nursing care. Protocol: Positioning of colostomy before surgery, close observation of vital signs, psychological nursing intervention, instruction of correct nursing methods, and enhancement in patients’ self-care ability.	Compared to the preoperative period, the SAS scores were significantly improved after comprehensive nursing care, which was superior to the control group (P < 0.05). This comprehensive care could promote postoperative recovery, decline the postoperative complications, and improve quality of life.
(25) Netherlands	Prospective monocenter study	50 patients	Colorectal cancer	NA	Implementing perioperative music. Protocol: A tailored implementation strategy of perioperative music intervention by calculating a median knowledge score.	Postoperative anxiety scores were lower than that of preoperative period (4.5 vs 3.0 scores). Perioperative music intervention was effective in reducing perioperative anxiety. Identifying the facilitating factors for implementing music might be more prominent.
(30) China	RCT	100 patients	Colorectal cancer	STAI	Continuous nursing care. Protocol: WeChat, Tencent QQ, and Tencent conference software were applied. 1. The patients were encouraged to post daily self-care methods and photographs or videos of existing problems after surgery. 2. Video lectures on the latest progress in colostomy- or ileostomy-related treatment and the new ostomy products and introductions. 3. Nursing materials that help the patients return to social life.	Compared to the control group, patients under continuous care had significantly lower mean STAI scores (both state anxiety and trait anxiety), indicating lower levels of anxiety after interventions.

STAI, State-Trait Anxiety Inventory; HADS, Hospital Anxiety and Depression Scale; SAS, self-rating anxiety scale; HAMA, Hamilton anxiety scale.

**Table 2:** Characteristics of the included studies on gastric cancer.

Study/Reference	Study design	Sample size	Cancer type	Assessment for anxiety	Nursing methods and protocols	Main findings
(18) China	Retrospective study	128 patients	Gastric cancer	HADS	Video-based nursing education. Protocol: A 20-min video which showed the information of this surgery and hospitalization, including benefits of minimally invasive gastrectomy, team members, anesthesia, environment of waiting room, operating room and recovery room, fluid intake and postoperative care.	The prevalence of anxiety was significantly lower in the intervention group than the control group (59.4% vs 76.6%, P = 0.037). The anxiety level dropped dramatically at 24h after surgery in both groups. Consistently, the anxiety score and prevalence of patients with anxiety were lower in intervention group than the control group (all P <0.05).
(19) China	Cohort	128 patients	Gastric cancer	SAS	Multidisciplinary cooperative continuous care. Protocol: 1. Establishment of a multidisciplinary cooperative continuous nursing group; 2. Specific interventions: distribution of nursing services, scheduled plan, data collected for establishing continuous nursing files, WeChat for communication, telephone follow-up, home-visit, and health education lectures.	The SAS score in the study group was significantly lower than that in the control group (47.14 ± 5.40 vs. 51.13 ± 5.09, P<0.001). Multidisciplinary cooperative continuous nursing could also ameliorate the depression, postoperative pain, and quality of life.
(21) China	Case control	88 patients	Gastric cancer	HAMA	Accelerated rehabilitation nursing. Protocol: 1. Preoperative nursing: explanation the surgery, 6h of fasting and water deprivation, and glucose water 2 h before surgery; 2. Intraoperative nursing: kept warm and controlled infusion; 3. Postoperative nursing: pain nursing, psychological nursing, exercise rehabilitation nursing; 4. Promote bowel movement.	HAMA score in the study group after intervention were significantly lower than that of the control group (P<0.05). Accelerated rehabilitation nursing also improved postoperative recovery of intestinal function, nutritional status, the quality of life, and reduced the incidence of complications.
(24) China	RCT	103 patients	Gastric cancer	HAMA	Traditional Chinese medicine (TCM) nursing. Protocol: The study group was received TCM nursing until the patients were discharged. The methods included TCM psychological care, syndrome differentiation nursing, dietetic nursing, and nursing of auricular-plaster therapy of TCM.	After TCM nursing intervention, the scores of HAMA were significantly lower than those before intervention (7.69 ± 1.29 vs 5.90 ± 1.23, P<0.05). HAMA scores in the study group were remarkable lower than those in the control group (5.90 ± 1.23 vs 6.80 ± 1.11, P<0.05). Perioperative TCM nursing could improve postoperative gastrointestinal dysfunction, alleviate acute inflammation, reduce postoperative complications, and improve the quality of life of postoperative patients.
(31) China	Retrospective study	100 patients	Gastric cancer	SAS	Evidence-based early warning nursing. Protocol: 1. Before surgery, the patient’s disease condition and psychological state were evaluated. 2. Targeted psychological counseling to help them to adjust their psychological state. 3. One day before surgery, the patients were introduced the anesthesia purpose, method, requirements and possible discomfort of the surgery. 4. Effective intraoperative nursing to avoid the occurrence of intraoperative accidents. 5. Early warning of physiological, drug, and psychological care for patients after surgery.	Patients received evidence-based early warning nursing exerted a significantly declination of SAS scores than that in the routine group (P<0.001). Additionally, under this nursing model, nurses could improve the care plans that reduce postoperative complications and pain for the patients, which might enhance the patient satisfaction.

STAI, State-Trait Anxiety Inventory; HADS, Hospital Anxiety and Depression Scale; SAS, self-rating anxiety scale; HAMA, Hamilton anxiety scale.

**Table 3:** Characteristics of the included studies on other GI cancers.

Study/Reference	Study design	Sample size	Cancer type	Assessment for anxiety	Nursing methods and protocols	Main findings
(28) China	Cohort	60 patients	Liver cancer	SAS	Comprehensive Nursing Approach. Protocol: 1. Preoperative care: introduced hospital ward environment, on-site lectures on negative psychology, explanation of successful treatment cases, helping the reduce avoid vasospasm negative psychology. 2. Intraoperative care: Reduced the patient’s tension and avoid vasospasm. 3. Postoperative nursing: Taken rest in bed for 1 day after surgery and move their bodies slightly.	The SAS scores of patients in the comprehensive nursing group were significantly lower than that of the conventional group (P<0.05). In addition, comprehensive nursing approach ensure a smooth operation, improve postoperative quality of life, reduce postoperative adverse reactions, and enhancing patients’ satisfaction.
(29) China	Cohort	105 patients	Liver cancer	Zung SAS	Comprehensive nursing care Protocol: 1. Preoperative care: providing some information related to the surgery to bridge gap between nursing staff and patients. 2. Postoperative care: Communicating with patients about their pain and other adverse effects. Explaining and providing the overcoming measures of adverse effects.	Pre- and post-operative comprehensive nursing care relieved the anxiety status of the patients and improved the quality of life patients who underwent surgeries for liver cancer.
(26) China	Retrospective study	80 patients	Gallbladder cancer	HAMA	Target nursing care. Protocol: The interventions included medication care, diet care, health advice for admission, preoperative and postoperative care, psychotherapy and progressive muscle, relaxation therapy and instructions for the surgical management.	Patients with target nursing care showed significantly lower HAMA scores than the control group (P<0.01). Besides, the study group also showed more promising effects on depression than the control group (P<0.01). However, target nursing care did not significantly improve the quality of life before and after intervention.
(16) China	Cohort	155 patients	Esophageal cancer	SAS	High-quality nursing. Protocol: Psychological nursing: communication, solving patient’s problems, explaining the knowledge of esophageal cancer, precautions after operation and possible complications; Pain care: Keeping a comfortable posture, breathe deeply and relax, diverting patient’s attention by movie, music, and chatting; Nutritional nursing: applying Peptisorb, Nutrison, etc; Respiratory tract nursing; Nursing care of the incision; Nursing of respiratory function training.	The SAS scores of patients with high-quality nursing were significantly lower than the control group (P <0.05). Additionally, the pain scores and depressive scores were also remarkably decreased after interventions. Furthermore, high-quality nursing could reduce pain and adverse events and promote rehabilitation.

STAI, State-Trait Anxiety Inventory; HADS, Hospital Anxiety and Depression Scale; SAS, self-rating anxiety scale; HAMA, Hamilton anxiety scale.

## Colorectal cancer

Anxiety is one of the most common psychiatric disorders in preoperative patients with colorectal cancer. The prevalence of preoperative anxiety in patients with colorectal cancer among different studies ranged from 41.9% to 56% (32, 33). In this review, nine relevant studies reported the different nursing interventions on the perioperative anxiety in patients with colorectal cancer.

Therapeutic listening (also namely active listening), a communication resource, has been found to be an effective method for reducing psychological comorbidities, including anxiety (34, 35). This is characterized by a series of interactions between the professional and patient where the patient is allowed to express his/her concerns or apprehensions freely. In this process, the professional helps patients to decrease anxiety and increase their capacity to adapt. Even though listening is recognized as a therapeutic tool, few studies have been conducted on this subject. Garcia et al. (14) conducted a RCT aimed to investigate the effects of therapeutic listening intervention on state anxiety preoperative colorectal cancer patients. The protocol of therapeutic listening intervention was that patients had half-hour to talk to the investigators about their experience with hospitalization for cancer treatment in the preoperative period. The results of Garcia et al.’s study (14) demonstrated that therapeutic listening intervention as a nursing intervention did not cause differences on the state anxiety and surgical fears compared to the control group (without listening intervention) (all P>0.05).

In the recent years, non-pharmacological interventions have been found to play an essential role in perioperative nursing care of patients undergoing surgical procedures (36). It was reported that perioperatively applied music intervention might decrease the preoperative anxiety as well as reduce the postoperative pain (37). Kakar et al. (25) conducted a prospective mono-center study related to the music intervention in Netherlands. This study included 50 colorectal cancer patients with a median age of 62.5 years. Patients in the interventional group received implementing perioperative music, which conducted by a tailored implementation strategy of perioperative music intervention by calculating a median knowledge score. Postoperative anxiety scores were lower than that of preoperative period (4.5 vs 3.0 scores). Perioperative music intervention was effective in reducing perioperative anxiety. The authors further suggested that identifying the facilitating factors for implementing music might be more prominent. The affecting factors may be associated with the attitudes, perceptions, beliefs of patients, healthcare professionals, and culture of nurses regarding music intervention.

Jin et al. (15) performed a comparison of anxiety before and after colostomy surgery in patients with colorectal cancer by using the HADS. This study showed that nearly half of participants were identified to have anxiety disorder before the surgery, while the number decreased to 37.3% in postoperative period. The different was significant (P<0.05). Age and educational background were the significant variables that influenced anxiety before and after surgery. The authors concluded that nurses should ascertain and meet patients’ personalized psychosocial needs, which might help them improve their anxiety symptom as well as their psychosocial behavior reactions.

There is positive association between stress and cancer as well cancer-related treatments (38). Under stress situation, the hypothalamicpituitary-adrenal (HPA) axis and the sympathetic nervous system are activated, resulting a decline and dysfunction of the prefrontal cortex and the hippocampus (39). In addition, stress may also cause the suppression of the immune system and T-cells proliferation. Interestingly, relaxation techniques, such as progressive relaxation exercise (PRE), can reduce the levels of anxiety in postoperative patients with cancer. An important aspect of PRE is the ability to contract and relax large muscle groups in a systematic and voluntary way. Ozhanli et al. (27) designed a RCT study for arranging colorectal cancer patients to take the PRE for 15min preoperatively and on postoperative days 1, 2, and 3 after breathing exercise training. As compared to the routine care, PRE could significantly reduce postoperative pain, the rate of using opioid analgesic, and the anxiety levels on postoperative day 0 (all *P*<0.05). This study demonstrated that PRE might be an effective intervention that nurses could administer independently to reduce the anxiety status of patients after colorectal cancer surgery.

Colostomy is a conventional step in rectal cancer resection. Patients with colostomy have lower anastomotic leakage and less risk of reoperation than those without colostomies (40). However, the postoperative complications will elevate after colostomy, which can increase the risk of psychological disorders. Having undergone rectal cancer stoma surgery, patients are prone to experiencing negative emotions such as anxiety and depression. Recently, some studies indicate that the selection of the nursing measures before and after the operation may help to reduce such psychological illnesses (41). Predictive nursing is a new medical model which summarizes the characteristics of specific diseases and patients’ behaviors. Li et al. (17) recruited 130 rectal cancer patients and investigated the anxiety status by using SAS. Preoperatively, the patients and their families were educated about rectal cancer. Then, the patients’ psychological states were evaluated and were managed by some interventions to eliminate their anxiety. Multimedia mode was applied to explain stoma care, diet indexes, bathing mode, and exercise mode. The results demonstrated that the SAS scores were significantly lower in the predictive nursing group than the control group (P < 0.05). Li et al.’s cohort (17) indicated that a predictive nursing approach could improve patient anxiety and self-management abilities following a rectal cancer colostomy. In line with this finding, Wang et al. found that the SAS and HAMA scores in the study group (rectal cancer patients undergoing ostomy with psychological interventions in the nursing care) were significantly lower than the control group (conventional nursing) (all *P*< 0.05). The nursing protocol in this study included the following items: 1. Preoperative psychological care; 2. Provided regular cleaning stoma; 3. Guided the patients could manage their stomas independently; 4. Diet and nutrition education; 5. Individualized psychological intervention; 6. Home care was taught before the discharge. This study suggested that psychological interventions in the nursing care could alleviate the patients’ bad moods, improve the quality of life, elevate the sleep durations. A more recent RCT conducted by Hao et al. (30) also showed that continuous nursing care significantly decreased the mean STAI scores (both state anxiety and trait anxiety) in colorectal cancer patients with colostomy or ileostomy. The methods of this intervention applied several software, including WeChat, Tencent QQ, and Tencent conference. The patients were firstly encouraged to post daily self-care methods and photographs or videos of existing problems after surgery. The nurses used the video lectures on the latest progress in colostomy- or ileostomy-related treatment and the new ostomy products and introductions. In addition, nursing materials were also applied to help the patients return to social life.

Two included studies (22, 23) applied comprehensive nursing care aiming to reduce the anxiety level in patients undergoing colorectal cancer surgery. Wu et al. (22) investigated the changes of anxiety in 110 colon cancer patients with or without comprehensive nursing. The protocols included: 1. Preoperative nursing: understood the patient’s physical state, medical and surgical history, systematic health education and psychological counseling; 2. Intraoperative nursing: cooperated with physicians in performing surgery-related nursing during the surgery; 3. Postoperative nursing: set comfortable position after surgery, appropriate exercise, and rigorous care. The results showed that the HAMA scores in the study group were significantly lower than those in the control group (P<0.05). Comprehensive nursing could significantly reduce the postoperative complication rate (9% vs 25%), increase the gastrointestinal quality of life index (GIQLI) score, and improve the prognosis. Consistently, Yu et al.’s study (23) also confirmed the positive effect of comprehensive nursing on perioperative anxiety. Compared to the preoperative period, the SAS scores were significantly improved after comprehensive nursing care, which was superior to the control group (P < 0.05). This comprehensive care could promote postoperative recovery, decline the postoperative complications, and improve quality of life. The protocol of this study included positioning of colostomy before surgery, close observation of vital signs, psychological nursing intervention, instruction of correct nursing methods, and enhancement in patients’ self-care ability. The characteristics of aforementioned nine included studies were listed in **Table 1**.

## Gastric cancer

Preoperative psychological distress was found in 76.97% of patients with newly diagnosed gastric cancer (42). Surgical gastrectomy is still the most effective way to treat gastric cancer. However, gastric cancer patients may suffer from significant anxiety during the perioperative period (43). At present, multiple nursing interventions have been performed to alleviative perioperative anxiety in patients with gastric cancer. Within the topic of this review, five included studies reported that specific preoperative nursing could reduce perioperative anxiety. Liu et al. (18) reported that video-based nursing education could significantly alleviative perioperative anxiety in patients with gastric cancer. The nurses would display a 20-min video which showed the information of this surgery and hospitalization, including benefits of minimally invasive gastrectomy, team members, anesthesia, environment of waiting room, operating room and recovery room, fluid intake and postoperative care. This study reported that the prevalence of anxiety was significantly lower in the intervention group than the control group (59.4% vs 76.6%, P = 0.037). The anxiety level dropped dramatically at 24h after surgery in both groups. Consistently, the anxiety score and prevalence of patients with anxiety were lower in intervention group than the control group (all P <0.05).

Continuous nursing intervention is an extension of inpatient nursing model. Rui et al. (19) explored the effect of multidisciplinary cooperative continuous nursing on the anxiety gastric cancer patients. For this nursing intervention, the nurses conducted the establishment of a multidisciplinary cooperative continuous nursing group. The specific interventions included distribution of nursing services, scheduled plan, data collected for establishing continuous nursing files, WeChat for communication, telephone follow-up, home-visit, and health education lectures. The authors found that the SAS score in the study group (gastric cancer patients with continuous nursing) was significantly lower than that in the control group (47.14 ± 5.40 vs. 51.13 ± 5.09, *P*<0.001). Multidisciplinary cooperative continuous nursing could also ameliorate the depression, postoperative pain, and quality of life.

Reasonable rehabilitation nursing has been found to accelerate postoperative recovery in patients with gastric cancer. Besides, this novel nursing model can significantly reduce or block patients’ physiological stress during the perioperative period. In Wang et al.’s study (20), the protocols of accelerated rehabilitation nursing included four key-points, including: 1. Preoperative nursing: explanation the surgery, 6h of fasting and water deprivation, and glucose water 2 h before surgery; 2. Intraoperative nursing: kept warm and controlled infusion; 3. Postoperative nursing: pain nursing, psychological nursing, exercise rehabilitation nursing; 4. Promote bowel movement. The authors implied that HAMA score in the study group after exercise rehabilitation intervention were significantly lower than that of the control group (P<0.05). Accelerated rehabilitation nursing also improved postoperative recovery of intestinal function, nutritional status, the quality of life, and reduced the incidence of complications.

Traditional Chinese medicine (TCM) nursing is one of the nursing interventions implemented according to TCM syndrome differentiation nursing theory (44). TCM nursing is an effective clinic care after malignant tumor resection. In a previous RCT study, Zhang et al. (24) implied that the scores of HAMA after TCM nursing intervention were significantly lower than those before intervention (7.69 ± 1.29 vs 5.90 ± 1.23, *P*<0.05). HAMA scores in the TCM nursing group were remarkably lower than those of conventional nursing group (5.90 ± 1.23 vs 6.80 ± 1.11, *P*<0.05). The study group was received TCM nursing until the patients were discharged. The methods included TCM psychological care, syndrome differentiation nursing, dietetic nursing, and nursing of auricular-plaster therapy of TCM. This RCT study demonstrated that perioperative TCM nursing could improve postoperative gastrointestinal dysfunction, alleviate acute inflammation, reduce postoperative complications, and improve the quality of life of postoperative patients.

Anesthesia is an essential part of operation of cancer. High quality of anesthesia is proved to have positive influence on the physiology and psychology of cancer patients after surgery (45). With the emergence and development of evidence-based medicine, evidence-based nursing (EBN) has become a nursing model influenced by clinical practice (46). EBN is designed for formulating nursing plans according to common clinical nursing problems, which combined with clinical professional knowledge and patient needs. Zhou et al. (31) conducted a retrospective study that included 100 gastric cancer patients. They found that patients received evidence-based early warning nursing exerted a significantly declination of SAS scores than that in the routine group (P<0.001). Besides, under this nursing model, nurses could improve the care plans that reduce postoperative complications and pain for the patients, which might enhance the patient satisfaction. The strategy of nursing plans included the following aspects. First, before surgery, the patient’s disease condition and psychological state were evaluated. Second, targeted psychological counseling to help them to adjust their psychological state. Third, one day before surgery, the patients were introduced the anesthesia purpose, method, requirements and possible discomfort of the surgery. Fourth, effective intraoperative nursing to avoid the occurrence of intraoperative accidents. Fifth, early warning of physiological, drug, and psychological care for patients after surgery.

## Other GI cancers

In the eighteen included studies, only four of them reported the nursing interventions on anxiety in patients with other GI cancers in addition to colorectal cancers and gastric cancer. Two studies (28, 29) applied the comprehensive nursing approach to reduce the perioperative anxiety in patients with liver cancer. However, there were some differences in their protocols. The comprehensive nursing approaches in Yuan et al.’s study (28) included the following items. In the preoperative care, the nurses would introduce hospital ward environment, on-site lectures on negative psychology, explanation of successful treatment cases, helping the reduce avoid vasospasm negative psychology. For the intraoperative care, the clinicians and the nurses intended to reduce the patient’s tension and avoid vasospasm. In the postoperative nursing, the patients would be arranged to take rest in bed for 1 day after surgery and move their bodies slightly. Inconsistent to Yuan et al.’s study, the comprehensive nursing care in Chen et al.’s study (29) mainly included preoperative care (providing some information related to the surgery to bridge gap between nursing staff and patient and postoperative care (communicating with patients about their pain and other adverse effects; explaining and providing the overcoming measures of adverse effects). Yuan et al. (28) demonstrated that the SAS scores of patients in the comprehensive nursing group were significantly lower than that of the conventional group (P<0.05). In addition, comprehensive nursing approach ensure a smooth operation, improve postoperative quality of life, reduce postoperative adverse reactions, and enhancing patients’ satisfaction. Chen et al. found that pre- and post-operative comprehensive nursing care relieved the anxiety status of the patients and improved the quality of life patients who underwent surgeries for liver cancer.

A retrospective study developed by Liu et al. (26) explored the effects of target nursing care on perioperative anxiety in patients with gallbladder cancer. The nursing interventions included medication care, health advice for admission, diet care, preoperative and postoperative care, psychotherapy and progressive muscle, relaxation therapy and instructions for the surgical management. Patients with this target nursing care showed significantly lower HAMA scores than the control group (P<0.01). Besides, the study group also showed more promising effects on depression than the control group (*P*<0.01). However, target nursing care did not significantly improve the quality of life before and after intervention.

Chen et al. (16) investigated the effects of high-quality nursing on anxiety state in patients with esophageal cancer after radical resection. Such high-quality nursing included psychological nursing (communication, solving patient’s problems, explaining the knowledge of esophageal cancer, precautions after operation and possible complications), pain care (keeping a comfortable posture, breathe deeply and relax, diverting patient’s attention by movie, music, and chatting), nutritional nursing (applying Peptisorb, Nutrison, etc), respiratory tract nursing, nursing care of the incision, and nursing of respiratory function training. The results turned out that the SAS scores of patients with high-quality nursing were significantly lower than the control group (P <0.05). Besides, the pain scores and depressive scores were also remarkably decreased after interventions. This study concluded that high-quality nursing could reduce pain and adverse events and promote rehabilitation.

## Limitations

Though this is a comprehensive review on the topic of the managements for perioperative anxiety in patients with GI cancers, some limitations should be acknowledged. As shown in the **Tables 1**–**3**, only a few included studies provided the proportion of anxiety of the GI cancer patients before and after the intervention in the perioperative period, we therefore cannot perform a meta-analysis to combine the odds ratio (OR) or relative risk (RR) from each eligible study. Since this is not a meta-analysis, we fail to judge which intervention may be the best for perioperative anxiety in patients with GI cancers. However, we can note that 14 out of 18 included studies reported the anxiety scores were significantly improved after different interventions (all P<0.05). All the conditions for these specific interventions occurred during the preoperative or postoperative period. However, different geographic locations, patient characteristics, and hospital characteristics may have some impacts on the outcomes of these interventions.

## Future perspective

Based on the current evidence on this topic of this review, we have posted some recommendation for future research. First, further explore the development of personalized anxiety management plans based on individual patient characteristics such as age, gender, stage of cancer, and psychological resilience. Determine which combinations of therapies are most effective for different patient subgroups. Second, assess the impact of anxiety management on the compliance and success of postoperative treatment regimens, such as the subsequent chemotherapy and radiation therapy. Also, standardized anxiety assessment tools should be further confirmed by the guideline. Third, since this review showing that the short-term effects of the various managements for perioperative anxiety with UI cancers, the long-term effects of the aforementioned interventions are supposed to further investigation in the future. Fourth, strengthen the collaboration between oncologists, surgeons, psychologists, and nurses to develop comprehensive perioperative care plans that address anxiety from multiple perspectives. Fifth, explore the use of virtual reality, mobile apps, and online platforms for delivering anxiety management interventions. Evaluate their accessibility, effectiveness, and patient acceptance. Last, for the specific patient populations, it is suggested that design and evaluate educational programs for patients and their families on understanding perioperative anxiety and available management strategies. Determine the optimal timing and format of these programs. The roles of socioeconomic factors in perioperative anxiety should be exhaustively studied in the subsequent studies.

## Translation of this review into clinical practice

Integrative care for perioperative anxiety in patients with GI cancers refers to a comprehensive approach that combines multiple therapeutic modalities to address the anxiety experienced by these patients before and after surgery. Individual counseling sessions with psychologists or psychiatrists can help patients understand and manage their anxiety. Through talk therapy, patients can express their fears, concerns, and emotions related to the GI cancer diagnosis, surgery, and prognosis. Patients can learn relaxation techniques, positive self-talk, and problem-solving skills to cope with stress. Establishing support groups is also an effective way to manage perioperative anxiety. Facilitated by healthcare professionals, support groups can offer educational resources and opportunities for emotional support. In clinical practice, by implementing an integrative care approach that addresses the psychological aspects of perioperative anxiety in patients with GI cancers, the clinicians and nurses can help improve patient outcomes and quality of life.

## Conclusion

The present review highlighted that there is a high prevalence of perioperative anxiety in patients with GI cancers (i.e., colorectal cancer, gastric cancer, liver cancer, gallbladder cancer, and esophageal cancer), while specific interventions are the reliable methods to reduce postoperative anxiety. Chen et al.’s study (16) might be the representative cohort due it was the largest sample size study. This study demonstrated that the SAS scores of patients with high-quality nursing intervention were significantly lower than the control group. The pain scores and depressive scores were also remarkably decreased after interventions. Furthermore, it could reduce adverse events and promote rehabilitation of the patients. The interventional strategies include, but are not limited to, therapeutic listening intervention, implementing perioperative music, predictive nursing, progressive relaxation exercises, psychological interventions in the nursing care, comprehensive nursing, continuous nursing care, video-based nursing education, multidisciplinary cooperative continuous care, accelerated rehabilitation nursing, TCM nursing, evidence-based early warning nursing, target nursing care, and high-quality nursing. Some limitations should be acknowledged when interpret the findings from the included studies, such as small sample size, no standard nursing intervention, inconsistent anxiety assessment tool, and various confounding factors (i.e., age, comorbidity, study design, and socioeconomic status). Therefore, a well-designed multicenter RCT with large sample size is still warranted for the confirmation of nursing intervention for managing perioperative anxiety in patients with GI cancers. Based on the data from this review, reduced perioperative anxiety levels by the relevant interventions can lead to improved mental health and have a positive impact on the body’s physiological responses to surgery. Managing perioperative anxiety is an important aspect of providing comprehensive care for patients with GI cancers. Effective management of perioperative anxiety for these patients may lead to reduced healthcare resource utilization. Therefore, personalized interventions and integrative care could be implemented in clinical practice, which may have a profound impact on patient outcomes and healthcare systems.
